# Morpho-molecular characterization and phylogenetic analysis of the Heterakidae nematode isolated from free-range chickens in the Sulaymaniyah Province, Iraq

**DOI:** 10.14202/vetworld.2025.2460-2466

**Published:** 2025-08-26

**Authors:** Shadan Hassan Abdullah

**Affiliations:** Department of Microbiology, College of Veterinary Medicine, University of Sulaimani, Sulaymaniyah, Iraq

**Keywords:** *cytochrome c oxidase subunit 1* gene, free-range chickens, *Heterakis gallinarum*, morphology, phylogenetic analysis, polymerase chain reaction, Sulaymaniyah

## Abstract

**Background and Aim:**

*Heterakis gallinarum* is a parasitic nematode that commonly infects the ceca of domestic and wild gallinaceous birds, acting as a vector for *Histomonas meleagridis*, the causative agent of blackhead disease. Despite its significance in poultry health, molecular data from Iraq, particularly Sulaymaniyah province, remain limited. This study aimed to characterize the morphological and genetic features of *H. gallinarum* isolated from free-range chickens in Sulaymaniyah using mitochondrial *cytochrome c oxidase subunit 1* (*COX1*) gene sequencing.

**Materials and Methods:**

A total of 140 free-range adult chickens were purchased from local markets in Sulaimani city between January 2023 and October 2024. Birds were euthanized, and cecal contents were examined for *Heterakis* spp. Adult worms were morphologically identified under light microscopy. DNA was extracted from representative isolates, and the *COX1* gene was amplified using polymerase chain reaction (PCR) and sequenced. Phylogenetic analysis was conducted using the neighbor-joining method based on the Kimura-3 parameter model.

**Results:**

Out of 140 chickens examined, 51 (36.43%) were infected with *H. gallinarum*. Morphological analysis revealed typical features, including unequal spicules in males and barrel-shaped eggs. PCR confirmed a 1325-bp *COX1* gene fragment. Basic Local Alignment Search Tool analysis showed 98%–99% similarity with known *H. gallinarum* sequences. Phylogenetic reconstruction clustered the Iraqi isolate (GenBank: PQ395216.1) with isolates from China, South Africa, Tunisia, and Bangladesh, indicating low genetic divergence across geographic regions.

**Conclusion:**

This is the first molecular documentation of *H. gallinarum* in free-range chickens from Sulaymaniyah, Iraq. The findings highlight the widespread genetic conservation of this parasite and underscore the need for further epidemiological studies to determine risk factors and potential impacts on poultry productivity.

## INTRODUCTION

*Heterakis* nematodes are globally distributed parasites that inhabit the ceca of various gallinaceous birds, including both wild and domesticated species [1]. Belonging to the family Heterakidae, order Ascaridida, and phylum Nematoda, several species have been identified within the genus *Heterakis*, such as *Heterakis* *gallinarum*, *Heterakis dispar*, *Heterakis indica*, *Heterakis isolonche*, *Heterakis beramporia*, *Heterakis papillosa*, *Heterakis dahomensis*, and *Heterakis spumosa* [2]. Among these, *H. dispar*, *H. isolonche*, and *H. gallinarum* are known to cause pathological lesions in the ceca of domestic and wild birds [3]. *H. gallinarum* is the most commonly reported species, especially in birds raised on soil or litter flooring systems [4], and has been documented in chickens, turkeys, pheasants, grouse, guinea fowl, partridges, ducks, geese, and quail [5].

*H. gallinarum* are small, slender, white roundworms, typically measuring 8–15 mm in length [6, 7]. Both sexes possess three prominent lips surrounding a slightly depressed anterior mouth. In males, the spicules are unequal in length, with the left spicule measuring 0.83–0.96 mm and the right 0.40–0.57 mm. The male also has a posteriorly tapering tail and a spherical, well-developed precloacal sucker encircled by a chitinized ring [8].

The length and symmetry of the spicules serve as critical morphological features for distinguishing *Heterakis* species, given that females appear morphologically similar across species. For instance, *H. dispar* has short, equal spicules; *H. gallinarum* has unequal spicules; and *H. isolonche* features long, nearly equal spicules [9].

Although infections with *H. gallinarum* often result in mild pathology and limited impact on poultry productivity [1], more severe infections may lead to petechial hemorrhages and bloody cecal exudates [1]. These nematodes exhibit exploratory behavior, feeding on tissue and exudates [10], and may cause nodular typhlitis [11]. Co-infections with *Histomonas meleagridis* can exacerbate the severity of lesions. High-density rearing conditions and litter-based systems increase the risk of parasite accumulation [1].

*Heterakis* nematodes have a direct life cycle and do not require an intermediate host [1]. Fertilized eggs are shed in feces and become infective in soil within approximately 2 weeks. Transmission occurs through ingestion of embryonated eggs or paratenic hosts such as earthworms [12]. Due to their resilience, *H. gallinarum* eggs can remain viable in the environment for over a year. Infected earthworms can also retain viable larvae for extended periods [13].

These worms are of economic significance because their eggs serve as a vehicle for *H. meleagridis*, the protozoan responsible for histomoniasis or blackhead disease, which is associated with high mortality rates in poultry [14, 15]. As long as the eggs remain viable, they can harbor *H. meleagridis* and release the protozoan on ingestion by a new host [13].

Molecular tools, particularly DNA markers, have proven effective in identifying and characterizing the genetic diversity of nematodes [16, 17]. Among these, the mitochondrial *cytochrome c oxidase subunit 1* (*COX1*) gene is widely used in taxonomic and phylogenetic studies across various parasitic nematodes [18, 19].

Backyard chickens are especially prone to infection due to their scavenging behavior, which increases environmental exposure to parasite eggs [20]. The parasite’s life cycle is perpetuated when chickens come into contact with their own feces [21], resulting in ongoing reinfection [1]. Environmental factors also influence transmission, for example, non-embryonated eggs develop more rapidly under fluctuating temperatures (12°C–22°C) than at a constant 15°C. Anaerobic conditions inhibit embryonation, likely due to the oxygen dependency of larval development [22].

Control measures include the use of anthelmintics, environmental disinfection, and management practices that limit exposure to contaminated areas. Resting poultry areas to allow natural egg degradation is also recommended [23].

Despite the global distribution and recognized significance of *H. gallinarum* as a parasitic nematode in poultry, data on its molecular characterization and phylogenetic relationships in the Middle East, particularly Iraq, remain scarce. Most existing literature focuses on morphological identification and prevalence rates in isolated geographic regions, with limited integration of genetic data to assess strain variability. Moreover, while mitochondrial genes such as *COX1* have been widely utilized in other parasitic nematodes for species differentiation and phylogenetic reconstruction, few studies have applied this molecular approach to *Heterakis* species in Iraq. Sulaymaniyah province, with its significant population of free-range chickens and traditional backyard farming practices, presents a potential hotspot for parasite transmission, yet no molecular epidemiological studies have been conducted in this area. This lack of region-specific genetic data hinders our understanding of *H. gallinarum* population structure, transmission dynamics, and evolutionary relationships within a broader global context.

To address these gaps, the present study aimed to investigate the occurrence and characterize *H. gallinarum* infecting free-range chickens in Sulaymaniyah province, Iraq, using an integrated morpho-molecular approach. Specifically, the study sought to (1) determine the prevalence of *H. gallinarum* in free-range chicken, (2) describe the morphological features of adult worms based on established taxonomic keys, (3) amplify and sequence a fragment of the mitochondrial *COX1* gene, and (4) analyze the phylogenetic relationship of the Iraqi isolate with reference strains available in GenBank. This study provides the first molecular record of *H. gallinarum* from Sulaymaniyah and contributes novel genetic data to the global repository, supporting future epidemiological surveillance, control strategies, and taxonomy of poultry nematodes.

## MATERIALS AND METHODS

### Ethical approval

The study was performed following bird decapitation; its flesh was used for consumption, and its intestine was used for parasitic detection. The approval was obtained from the Ethics committee of the College of Veterinary Medicine, University of Sulaimani (Approval No. VMUS.EC. Doc 9-2023). The study followed Animal Research: Reporting of *in vivo* Experiments (ARRIVE) guidelines.

### Study period and location

The study was conducted from January 2023 to October 2024 in Sulaymaniyah province, northern region of Iraq.

### Sample collection

A total of 140 adult free-range chickens were purchased from local markets in Sulaimani City. These chickens originated from multiple villages across the Sulaymaniyah province, located in the northern region of Iraq.

### Parasitological examination

On arrival, chickens were humanely euthanized through decapitation. Postmortem examinations were performed immediately, during which the ceca of each bird were dissected and examined for the presence of *Heterakis* nematodes. Recovered worms were collected, rinsed in sterile normal saline to remove debris, and preserved in 10% ethanol for further analysis.

### Morphological identification of nematodes

The preserved adult worms were examined under a compound microscope using a 10× objective lens. Morphological identification was conducted based on standard nematode taxonomic characteristics, following the criteria described by Soulsby [24]. Key features such as body shape, spicule length, presence of precloacal sucker, and cuticular structures were assessed to differentiate species.

### DNA extraction and *COX1* gene amplification

Genomic DNA was extracted directly from individual adult worm specimens using a commercial DNA extraction kit (Add-bio, Korea), in accordance with the manufacturer’s protocol. Amplification of the mitochondrial *COX1* gene was performed using published primer sets:

- *COX1*-F: 5′-TTTCA TACAGAATAAATATCAGGA-3′
- *COX1*-R: 5′-AGTTCTAATCAT AAGGATATTGGGA-3′.

These primers target a 1325 bp fragment of the *COX1* gene, conserved among *Ascaridia galli* and related nematodes [15]. Polymerase chain reaction (PCR) was carried out in 0.2 mL tubes containing a 20 μL reaction mixture: 10 μL of 2× SuPrime Script Premix (Genetbio, South Korea), 5 μL of template DNA, 1 μL of each primer (10 pmol), and 3 μL of nuclease-free water. Amplification was conducted using a BIO-RAD thermal cycler (USA) under the following conditions:

- Initial denaturation at 95°C for 5 min
- 40 cycles of denaturation at 95°C for 30 s
- Annealing at 59°C for 30 s
- Extension at 72°C for 60 s
- Final extension at 72°C for 5 min.

The PCR products were analyzed through electrophoresis on 1% agarose gels stained with Safe Dye (Gendirex, Tiwan) and visualized using an ultraviolet transilluminator (Syngene, UK) after running at 120 V for 60 min.

### Sequencing and GenBank accession

A representative PCR product was purified and sent for sequencing at Macrogen Inc. (Seoul, South Korea). The resulting *COX1* sequence was submitted to the National Center for Biotechnology Information GenBank database and assigned the accession number PQ395216.1, confirming its identity as *H. gallinarum*.

### Phylogenetic and sequence analysis

The *COX1* sequence obtained in this study was compared to reference *H. gallinarum* sequences available in the GenBank database using Basic Local Alignment Search Tool (BLAST). Multiple sequence alignments were performed using the Clustal-W (https://www.genome.jp/tools-bin/clustalw) algorithm. A phylogenetic tree was constructed in MEGA X software employing the neighbor-joining method based on the Kimura-3 parameter model [25]. Genetic distances among sequences were calculated, and the robustness of the phylogenetic clustering was evaluated using a bootstrap analysis with 100 replicates [26].

## RESULTS

### Prevalence of *H. gallinarum* in free-range chickens

Out of the 140 free-range chickens examined from the Sulaymaniyah province, 51 were found to be infected with *H. gallinarum*, yielding an overall prevalence rate of 36.43% (Table 1). *Heterakis* nematodes were observed within the cecal lumen on necropsy.

**Table 1:** Frequency of *Heterakis* nematodes isolated from the ceca of free-range chickens in the Sulaymaniyah province.

No. of chickens examined	Positive (%)	Negative (%)
140	51	36.43

### Morphological features of recovered worms

**Figure 1:**
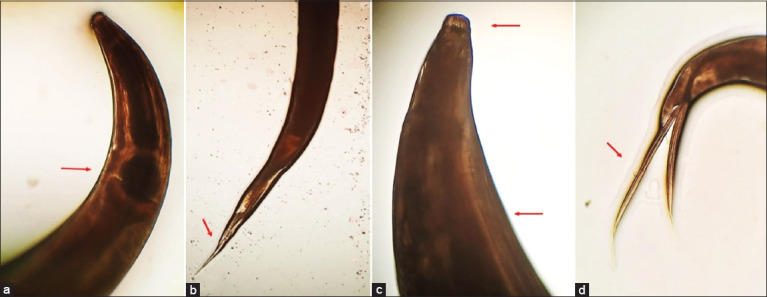
Morphological characteristic of *Heterakis* worms isolated from chickens: (a) The head of the female (revealing esophageal posterior bulb), (b) caudal end of the female (slender and pointed), (c) head of the male (revealing lips and lateral alae), and (d) caudal end of the male (with two copulatory spines of unequal length).

**Figure 2:**
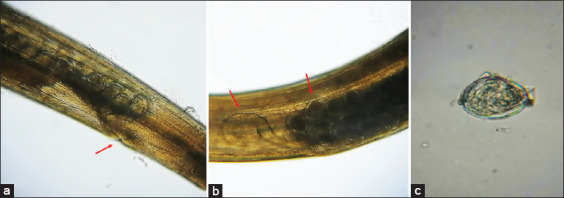
Morphological features of *Heterakis* nematodes isolated from chickens: (a) Female valval area, (b) mid portion of female illustrate vaginal bends and uterus with barrel shaped eggs, and (c) egg of *Heterakis* worm, oval-shaped with smooth, thick shell.

Microscopic examination of the recovered nematodes revealed distinct morphological features consistent with *H. gallinarum*. Male worms displayed unequal spicules and a well-defined precloacal sucker, while female worms exhibited a narrow-pointed tail and centrally located vulva. Detailed morphological structures of both sexes are illustrated in Figures 1 and 2.

### Molecular confirmation of *H. gallinarum*

**Figure 3:**
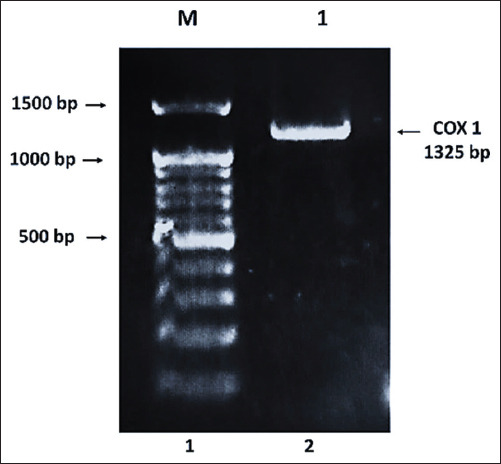
Molecular identification of the *Heterakis gallinarum* nematode, Lane M: DNA Marker, Lanes 1: *H. gallinarum* from ceca of free-range chicken.

Molecular analysis through PCR amplification confirmed the presence of *H. gallinarum*. A specific 1325-bp amplicon of the mitochondrial *COX1* gene was successfully amplified from the DNA of representative worms (Figure 3). This amplicon corresponds to the expected product size targeted by species-specific primers.

### Phylogenetic analysis and sequence identity

Phylogenetic reconstruction using the neighbor-joining method based on the *COX1* gene sequence revealed that the isolate from this study (GenBank accession no. PQ395216.1) shared 98%–99% sequence identity with previously characterized *H. gallinarum* isolates in the GenBank database.

### Clustering with global isolates

The phylogenetic tree demonstrated that *H. gallinarum* isolates formed two major clusters. The current isolate (PQ395216.1) was placed in Cluster 2, aligning closely with previously reported isolates from China (KP308348.1), South Africa (OL457523.1), Tunisia (MF066720.1, MF066713.1), and Bangladesh (LC592866.1), indicating low genetic diversity across geographically distinct populations.

## DISCUSSION

### Prevalence comparison across regions

Heterakidiosis in poultry has been reported in several countries, with varying prevalence rates depending on region, husbandry conditions, and climatic factors. In the present study, *H. gallinarum* infection was detected in 36.43% of free-range chickens examined in Sulaymaniyah Province. This prevalence rate is higher than previously reported rates from other regions, including 24.18% in India [27], 15.38% in Egypt [28], and 13.5% in Ethiopia [29]. However, it remains lower than the exceptionally high prevalence of 81% reported in a prior study from Iraq [30]. Notably, a similar infection rate of 36.66% was observed in turkeys in Egypt [12], indicating that free-range birds raised under comparable environmental and management conditions may exhibit parallel levels of exposure and susceptibility. These differences in prevalence may be attributed to local environmental conditions, hygiene practices, poultry density, and deworming protocols.

### Factors influencing transmission and persistence

The incidence of *Heterakis* infection is influenced by multiple ecological and management-related factors. *H. gallinarum* eggs are known for their environmental resilience, remaining viable for up to 3 weeks under favorable conditions. Transmission is facilitated through fecal-oral routes, with contamination of water sources, feed, and soil. Mechanical vectors such as houseflies and biological vectors like earthworms can act as paratenic hosts, further propagating infection [31]. In addition, poor nutritional status, particularly Vitamin A deficiency, compromises host immunity, increasing susceptibility to parasitic infections. Overcrowded conditions and inadequate sanitation further compound the risk of reinfection [32].

### Control and therapeutic strategies

Effective control of heterakidiosis requires a multifaceted approach, integrating good husbandry practices with appropriate anthelmintic therapy. Interrupting the parasite’s life cycle is critical, particularly in settings where reinfection is common. Regular cleaning of poultry housing, rest periods for contaminated outdoor areas, and provision of clean feed and water can significantly reduce infection pressure. Pharmacologically, benzimidazoles, especially flubendazole (Flimabend, USA), are routinely used in poultry to treat *A. galli* and *Heterakis* spp. infections [33]. However, concerns regarding drug resistance highlight the importance of rational and evidence-based deworming schedules.

### Morphological confirmation of *H. gallinarum*

Morphological analysis of the recovered worms corroborated their identification as *H. gallinarum*. The nematodes appeared thin, creamy white, and sexually dimorphic. Females were longer than males, and both sexes exhibited three well-defined, equally sized lips at the anterior end. The esophagus terminated in a prominent bulb. Males displayed lateral alae and subequal spicules with a distinctive curved-up tip (Figure 1). Females exhibited a narrow, pointed tail, a vulval opening located at mid-body, and a visible vaginal bend (Figures 1 and 2). These features are consistent with descriptions of *H. gallinarum* reported in previous literature by Wangelu *et al*. [34] and El-Saied *et al*. [35].

### Genetic characterization and phylogenetic relationships

**Figure 4:**
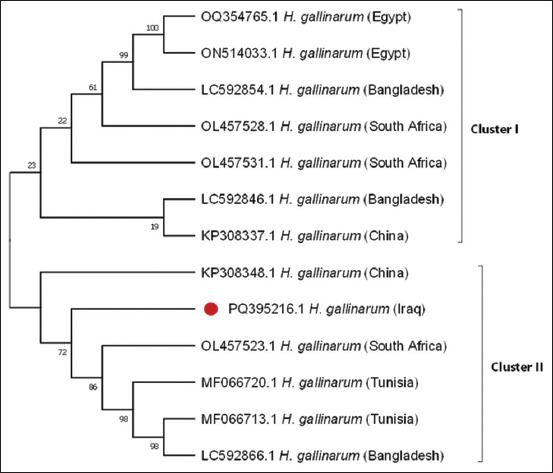
Phylogenetic relationships of *Heterakis* sequence isolates from the ceca of free-range chickens in Sulaymaniyah province with previously reported *Heterakis* isolates in the GenBank database were established. The tree was constructed based on *cytochrome c oxidase subunit 1* gene sequences using the neighbor-joining approach with the Kimura-3 parameter model in MEGA X software. The branching reliability was assessed through bootstrap analysis with 100 replicates.

The genetic identity of the recovered worms was further confirmed through *COX1* gene sequencing. BLAST analysis of the amplified 1325-bp fragment revealed 98%–99% sequence similarity to previously characterized *H. gallinarum* isolates. Phylogenetic analysis (Figure 4) positioned the current isolate (GenBank accession no. PQ395216.1) in close genetic proximity to isolates from China (KP308348.1) [15], South Africa (OL457523.1) [36], Tunisia (MF066720.1, MF066713.1) [37], and Bangladesh (LC592866.1) [38]. This clustering pattern suggests low genetic diversity among *H. gallinarum* populations across geographically distinct regions, potentially reflecting evolutionary conservation within the species.

## CONCLUSION

This study provides the first morpho-molecular confirmation of *H. gallinarum* infection in free-range chickens from Sulaymaniyah Province, Iraq, with an overall prevalence rate of 36.43%. Morphological examination revealed distinct species-specific features, including unequal spicules in males and characteristic anterior and tail structures in both sexes, consistent with established descriptions of *H. gallinarum*. Molecular validation through amplification of a 1325-bp fragment of the mitochondrial *COX1* gene further confirmed species identity. The Iraqi isolate (GenBank: PQ395216.1) showed 98%–99% sequence similarity with globally reported isolates and clustered phylogenetically with sequences from China, South Africa, Tunisia, and Bangladesh, indicating low genetic variability despite geographical separation.

The detection of *H. gallinarum* in backyard poultry systems has important implications for disease control and productivity. As a known vector for *H. meleagridis*, this parasite poses a potential threat to poultry health, particularly in free-range settings where environmental exposure and reinfection are common. The findings support the need for enhanced awareness among poultry keepers, implementation of targeted anthelmintic treatment programs, and improvements in environmental hygiene and flock management.

A key strength of this study is the integration of morphological and molecular approaches for accurate parasite identification, supported by comparative phylogenetic analysis. Moreover, this research fills a significant data gap, contributing the first molecular record of *H. gallinarum* in this region to global databases. However, the study is limited by its relatively small sample size and confinement to a single geographical location. The use of only one genetic marker (*COX1*) also restricts insight into broader genomic diversity. In addition, host-related and environmental factors influencing infection dynamics were not evaluated.

Future studies should include broader geogra-phic sampling, seasonal monitoring, and analysis of co-infections with *H. meleagridis* and other gastrointestinal parasites. Expanding genetic profiling to include additional loci could offer deeper resolution of strain diversity. Investigations into anthelmintic resistance and effectiveness of commonly used deworming protocols are also warranted.

This study presents important epidemiological and genetic insights into *H. gallinarum* infections in free-range chicken in Iraq. The results highlight the need for improved parasite surveillance, integrated control strategies, and sustained research to safeguard poultry health and rural livelihoods.

## AUTHOR’S CONTRIBUTIONS

SHA: Designed the study and contributed to sample collection, laboratory work, and data analysis. Literature collection and manuscript writing. The author has read and approved the final manuscript.
